# Association between previous surgical termination of pregnancy and pregnancy outcomes in frozen-thawed embryo transfer cycles of IVF/ICSI: a retrospective cohort study

**DOI:** 10.1038/s41598-024-57377-4

**Published:** 2024-03-19

**Authors:** Shuai Zhang, Hanhan Tang, Minglian Zhou

**Affiliations:** 1 Clinical Center of Reproductive Medicine, Lianyungang Maternal and Child Health Hospital, Qindongmen Avenue, Haizhou District, Lianyungang City, 22200 China; 2 Graduate School of Xuzhou Medical University, Xuzhou Medical University, No. 209, Tongshan Road, Xuzhou City, 221004 China

**Keywords:** Freeze–thaw embryo transfer, Induced termination of pregnancy, Induced abortion, Endometrium, Infertility, Infertility, Risk factors

## Abstract

This study aimed to evaluate the effect of previous surgical termination of pregnancy (STP) on pregnancy outcomes in women undergoing FET cycles of IVF/ICSI. Retrospective cohort study. Reproductive Center of Maternal and Child Health Care Hospital in Lianyungang city. Data were selected from all IVF/ICSI FET cycles performed between January 2014 and December 2020. A total of 761 cycles met the criteria were included in this study. The primary outcome measures were clinical pregnancy and live birth rates. Secondary outcome measures were biochemical pregnancy rate, spontaneous abortion rate, and preterm birth rate. After adjustments for a series of potential confounding factors, the previous STP was an influential factor in reducing FET cycle clinical pregnancy rate compared with women who had not previously undergone STP (OR = 0.614, 95% CI 0.413–0.911, P = 0.016). The effect of the previous STP on the live birth rate was not statistically significant. (OR = 0.745, 95% CI 0.495–1.122, P = 0.159). Also, an increase in the number of previous STPs relative to only 1-time abortion was an independent risk factor in reducing clinical pregnancy rate and live birth rate (OR = 0.399,95% CI 0.162–0.982, p = 0.046; OR = 0.32,95% CI 0.119–0.857, p = 0.023). Previous STP was an independent factor contributing to the decline in FET cycle clinical pregnancy rates.

## Introduction

Induced abortion is also called induced termination of pregnancy (ITP) is the intentional termination of a pregnancy using medical or surgical methods within the first three months of gestation^1^. A total of 121 million unplanned pregnancies were reported between 2010 and 2014 worldwide, of which 61% resulted in ITP^2^. In China, ITP is a typical response to unintended pregnancies. According to the 2021 China Health Statistics Yearbook, more than 8.9 million termination of pregnancy took place in China that year, with surgical termination of pregnancy (STP) accounting for the majority^3^. Generally, STP is performed in the first trimester of pregnancy. Vacuum aspiration (VA) has been adopted in many countries due to its superior efficacy, safety, sharp curettage, and dilatation and evacuation (D&E)^1^. Infection, bleeding, perforation, acute hematoma, and tissue retention are typical early complications of ITP^1,4^. A rise in stillbirths and secondary infertility are examples of late complications of ITP^5–8^. A total of 48 studies published by the World Health Organization (WHO) reported that the incidence of secondary infertility due to ITP ranged from 1 to 7.6%^9^. Possible causes of secondary infertility include pelvic infection after ITP and changes in endometrial receptivity^6,10,11^.

Infertility affects approximately 186 million people universally as a global disease^12^. South and Central Asia, Sub-Saharan Africa, North Africa/Middle East, and Central/Eastern Europe have the highest infertility rates^13^. Moreover, these regions have a relatively high prevalence of unsafe abortion^14^. In vitro fertilization/intracytoplasmic sperm injection (IVF/ICSI) is widely used as an advanced technique for treating complex infertility. Studies have demonstrated that women who have previously undergone an abortion have thinner endometrium and poorer pregnancy outcomes in IVF cycles than those who have not^15,16^. This suggests that a history of ITP is one of the risk factors for poor pregnancy outcomes in IVF/ICSI cycles. With technological advancements, freeze–thaw embryo transfer (FET) is extensively used as it offers high pregnancy rates. This technique effectively prepares the endometrium, improves the endometrium receptivity, facilitates the rational use of gonadotropins (Gn), and reduces the occurrence of ovarian hyperstimulation syndrome^17^. Considering the advantages of FET compared to fresh cycle transplants, does the history of STP have an adverse impact on pregnancy outcomes during the FET cycle? As far as we know, there has been no research to answer this question. Therefore, we designed this study to assess the impact of previous STP on pregnancy outcomes in women treated with FET.

## Materials and methods

### Study population and design

All data were obtained from the electronic case system of the reproductive Medicine Center of Maternal and Child Health Hospital of Lianyungang city. We reviewed data from all IVF/ICSI FET cycles performed between January 2014 and December 2020.

Inclusion criteria were as follows, women aged ≤ 42 years, infertility duration ≥ 12 months, and all cycles of FET treatment. STP is defined as surgical termination of pregnancy, including STP performed for medical reasons (such as STP due to unavoidable abortion). The interval between STP and IVF was at least one year. Exclusion criteria were reproductive organ malformations (single horn uterus, double horn uterus, etc.), endometriosis, adenomyosis, intrauterine adhesions; chromosomal abnormalities in either spouse; previous medical abortion history; and patients with surgical miscarriage after the third month of pregnancy.

Only the first FET treatment cycles were included per couple to avoid cases with multiple repeat cycles involving the same couple.

To evaluate the effect of a prior STP on FET cycle pregnancy outcomes, we divided study participants into two groups based on whether patients had previously undergone a STP. STP group: women who had received FET and had previously undergone surgically induced pregnancy termination for non-medical reasons. Control group: women who received FET but did not experience ITP. Furthermore, we analyzed different subgroups: (i) To evaluate the difference in pregnancy outcomes at different ages, and the study participants were further divided into two subgroups based on their age: < 35 years old and ≥ 35 years old. (ii) To evaluate the influence of STP on pregnancy outcomes, the STP group was further divided into two subgroups based on the number of STP: 1-time and ≥ 2-times groups.

### Ethics approval and consent to participate

This study was approved by the Ethics Committee (Institutional Review Board) of Lianyungang Maternal and Child Health Hospital (approval ID. LW2022009). Written informed consent was waived by the Ethics Committee (Institutional Review Board) of Lianyungang Maternal and Child Health Hospital due to the retrospective nature, and patients' data were used anonymously. All methods were carried out in accordance with relevant guidelines and regulations.

### Patient and public involvement

None.

### Primary and secondary outcome measures

The primary outcome measures were clinical pregnancy and live birth rates during the FET cycle. Clinical pregnancy was defined as a pregnancy in which the gestational sac can be observed via TVUS at seven weeks. Clinical pregnancy rate = the number of clinical pregnancy cycles/total number of FET treatment cycles. Live birth was defined as a baby born alive after 28 weeks of gestation. Live birth rate = the number of live birth cycles/total number of FET treatment cycles.

Secondary outcome indicators were biochemical pregnancy rate, spontaneous abortion rate, and premature birth rate. The serum human chorionic gonadotropin (HCG) test was conducted 12 days post-embryo transfer to assess biochemical pregnancy, with a threshold for confirmation set at a serum HCG level exceeding 25 U/L. Biochemical pregnancy rate = the number of biochemical pregnancy cycles/total number of FET treatment cycles. Terminating pregnancy under any circumstances after a confirmed clinical pregnancy was considered spontaneous abortion, with spontaneous abortion rate = the number of cycles of spontaneous abortion/number of pregnancy cycles. Preterm birth was defined as delivery between 28 and less than 37 weeks of gestation: preterm birth rate = the number of preterm births/number of live births.

The reproductive center staff obtained all clinical outcome information through routine telephone follow-up and accurately recorded it in the electronic medical record system.

### Covariates

We collected the following data bout participants from the case system as covariables: Age, body mass index (BMI) (BMI < 18.5/18.5–25/ ≥ 25 kg/m^2^), number of transplanted embryos, development stage of transplanted embryos (cleavage stage and blastocyst stage), endometrial thickness, endometrial morphology (A, B, and C), duration of infertility, previous STP, and endometrial preparation protocol.

### Sample size calculation

One of the primary outcome indicators, the live birth rate, might have been affected by the relatively small sample size, leading to inadequate statistical power. Consequently, the sample size was determined with a focus on the live birth rate to ensure adequate statistical power. Drawing from previous studies^16^ and clinical data from our center, the STP group exhibited a live birth rate of approximately 25%, while the Control group demonstrated a rate of approximately 35%. With a type I error set at α = 0.05 and aiming for over 80% statistical power with a 1:3 ratio in sample sizes between the two groups, 207 and 621 participants were required for the STP and Control groups, respectively. These calculations were performed using PASS (NCSS Inc., USA, 2017 edition).

### Statistical analysis

SPSS 26.0 (IBM, Inc.) was used for statistical analysis. Values were expressed as mean ± SD. For the data with normal distribution, the independent sample T-test was used for inter-group comparison. Mann–Whitney U test was used to evaluate the statistical difference of non-normally distributed continuous variables. χ2 test was used for comparison between groups. Fisher's exact probability method was used when the χ^2^ test was not met. Binomial logistic regression analyses was used to adjust confounding factors to calculate the odds ratios (OR) of previous STP on the FET cycle clinical pregnancy rate and live birth rate. P < 0.05 was defined as statistically significant.

## Results

### Baseline characteristics

**Figure 1:**
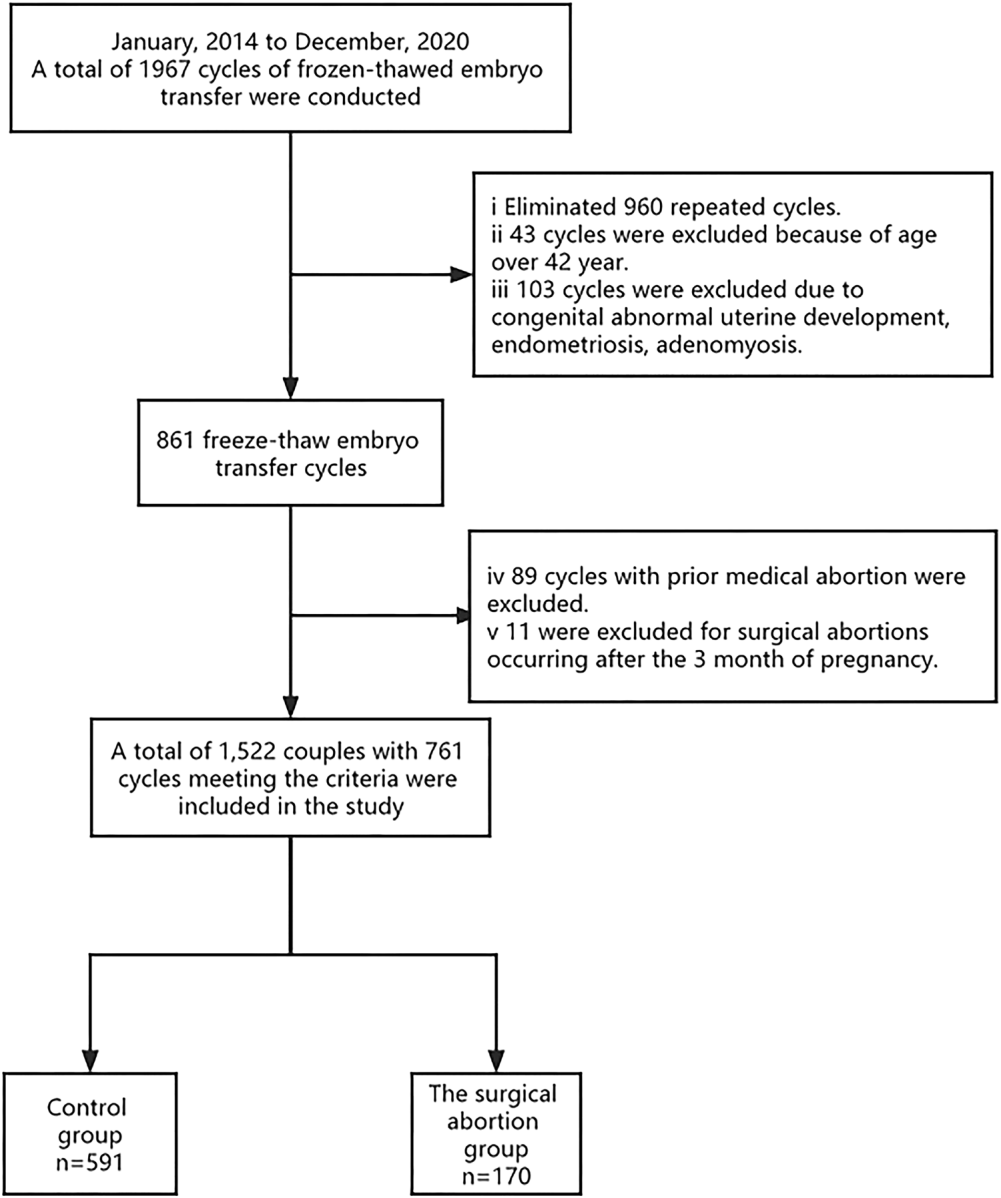
Flow chart of the study.

A total of 1967 FET cycles were conducted between January 2014 and December 2020. In total, 960 repeat cycles were excluded: 43 cycles were excluded because the age was over 42; 103 cycles were excluded due to congenital abnormal uterine development, endometriosis, or adenomyosis; 89 cycles with previous medical abortion were excluded, and 11 were excluded for STP occurring after the third month of pregnancy. A total of 761 cycles met the criteria were included in this study. In 761 cycles, 170 (22.3%) cycles had undergone STP, of which 121 (71.2%) had undergone one previous STP and 49 (28.8%) had undergone more than two STP previously, as demonstrated in Fig. 1.

Table 1 illustrates the baseline data of the STP group and the control group. The results revealed no statistical significance in the duration of infertility, BMI, basic endocrine status, number of transplanted embryos, embryo stage, and endometrial preparation protocol between the two groups. The age difference between the two groups was significant; the age of the STP group was significantly higher than that of the control group. The endometrial thickness of the STP group was significantly thinner than that of the control group.

**Table 1:** Comparison of baseline data between the surgical termination of pregnancy group and the control group.

Variable	Control group n = 591	The STP group n = 170	*P* value
Age (years)	31.09 ± 4.61	32.15 ± 4.56	0.002
< 35 years	464 (78.5)	117 (68.8)	0.009
≥ 35 years	127 (21.5)	53 (31.2)	
Duration of infertility (years)	4.49 ± 3.2	4.12 ± 3.14	0.185
Infertility type, n (%)			
Primary infertility	344 (58.2)	0 (0)	< 0.001
Secondary infertility	247 (41.8)	170 (100)	
Number of deliveries	0.24 ± 0.51	0.52 ± 0.64	< 0.001
BMI (kg/m^2^)	23.5 ± 3.32	23.16 ± 3.36	0.245
< 18.5	18 (3)	8 (4.7)	0.498
18.5–25	394 (66.7)	115 (67.6)	
≥ 25	179 (30.3)	47 (27.6)	
Endometrial thickness (mm)	11.43 ± 1.94	10.44 ± 2.05	< 0.001
Endometrial classification, n (%)			
A	44 (7.4)	12 (7.1)	0.984
B	520 (88)	150 (88.2)	
C	27 (4.6)	8 (4.7)	
Day 3 FSH (mIU/mL)	7 ± 2.32	7.38 ± 2.75	0.268
Day 3 LH (mIU/mL)	5.41 ± 4.28	4.89 ± 3.22	0.191
Day 3 E2 (pmol/L)	180.41 ± 255.31	171.17 ± 129.85	0.281
Number of embryos transferred	1.55 ± 0.52	1.51 ± 0.55	0.321
Embryo stage, n (%)			
Cleavage stage	448 (75.8)	140 (82.4)	0.073
Blastocyst stage	143 (24.2)	30 (17.6)	
Endometrial preparation protocol, n (%)			
Natural cycle protocol	164 (27.7)	55 (32.4)	0.505
Hormone replacement protocol	346 (58.5)	93 (54.7)	
Ovulation stimulation protocol	81 (13.7)	22 (12.9)	

Mann–Whitney U-test was used for the statistics of FSH, E2, LH.

*BMI* body mass index, *FSH* follicle-stimulating hormone, *LH* luteinizing hormone, *E2* estradiol, *STP* surgical termination of pregnancy.

### Pregnancy outcomes

The biochemical pregnancy rate and clinical pregnancy rate in the control group were significantly higher than the STP group. The statistical power of the biochemical pregnancy rate was 87.2% and the clinical pregnancy rate was 84.05%. The live birth rate in the control group was significantly higher than that in the STP group, differences between the two groups were close to significance (P = 0.072). It should be noted that the statistical power of the live birth rate is low at 46.56% due to the relatively inadequate sample size. There was no significant difference in the spontaneous abortion rate and premature birth rate between the two groups, as illustrated in Table 2.

**Table 2:** Comparison of FET cycle pregnancy outcomes between the surgical termination of pregnancy group and the control group.

Variable	Control group n = 591	The STP group n = 170	*P* value
Biochemical pregnancy rate, n (%)	267 (45.2)	55 (32.4)	0.003
Clinical pregnancy rate, n (%)	248 (42)	51 (30)	0.005
Live birth rate, n (%)	207 (35)	47 (27.6)	0.072
Spontaneous abortion rate, n (%)	34 (13.7)	3 (5.9)	0.122
Preterm birth rate, n (%)	22 (10.6)	7 (14.9)	0.406

*STP* surgical termination of pregnancy.

### Subgroup analysis

#### Age stratification

The study participants were stratified according to their age: < 35 and ≥ 35 years old. The control group was divided into A1 and A2 groups, and the STP group was similarly divided into B1 and B2 groups. After stratification, we conducted intra-group and inter-group comparative studies, respectively, and the results displayed that: the endometrial thickness of the B2 group was significantly thinner than that of the B1 group. The endometrial thickness of the STP group was significantly lower than that of the control group (A1 vs. B1, A2 vs. B2, P < 0.001). The biochemical pregnancy rate, clinical pregnancy rate, and live birth rate in the group aged ≥ 35 years were significantly lower than those in the group aged < 35 years. Although the biochemical pregnancy rate, clinical pregnancy rate, and live birth rate in the STP group were lower than those in the control group (A1 vs. B1 and A2 vs. B2), the difference in clinical pregnancy rate was statistically significant only in the age group ≥ 35 years. Women aged ≥ 35 had a statistically higher rate of spontaneous abortion than the A1 and A2 groups. Although women aged ≥ 35 had a higher rate of spontaneous abortion than the B1 and B2 groups, the difference was not statistically significant (Table 3).

**Table 3:** Comparison of the general situation and FET pregnancy outcomes of the two groups divided into < 35 years and ≥ 35 years according to age.

	Control group n = 591		*P*^*a*^	The STP group n = 170		*P*^*b*^	*p*^*c*^	*p*^*d*^
	A1 < 35 years n = 464	A2 ≥ 35 years n = 127		B1 < 35 years n = 117	B2 ≥ 35 years n = 53			
Age (years)	29.2 ± 3	38 ± 2.25	< 0.001	29.68 ± 2.76	37.58 ± 2.61	< 0.001	0.111	0.315
Endometrial thickness (mm)	11.44 ± 1.96	11.38 ± 1.88	0.748	10.69 ± 2.02	9.9 ± 2.01	0.019	< 0.001	< 0.001
BMI (kg/m^2^)	23.36 ± 3.4	24.01 ± 2.98	0.051	22.67 ± 3.46	24.24 ± 2.85	0.005	0.052	0.630
Day3 FSH (mIU/mL)	6.89 ± 2.22	7.4 ± 2.62	0.020	7.15 ± 2.62	7.9 ± 2.98	0.163	0.503	0.561
Day3 LH (mIU/mL)	5.64 ± 4.64	4.56 ± 2.41	0.019	5.12 ± 2.99	4.38 ± 3.66	0.010	0.673	0.209
Day3 E2 (pmol/L)	174.87 ± 250.86	200.64 ± 271.01	0.306	169.53 ± 118.76	174.79 ± 152.71	0.639	0.159	0.829
Number of embryos transferred	1.58 ± 0.52	1.45 ± 0.53	0.013	1.53 ± 0.55	1.45 ± 0.54	0.396	0.360	0.963
Cleavage stage	339 (73.1)	109 (85.8)	0.003	91 (77.8)	49 (92.5)	0.020	0.298	0.216
Blastocyst stage	125 (26.9)	18 (14.2)		26 (22.2)	4 (7.5)			
Natural cycle protocol	122 (26.3)	42 (33.1)	0.301	38 (32.5)	17 (32.1)	0.997	0.408	0.965
Hormone replacement protocol	276 (59.5)	70 (55.1)		64 (54.7)	29 (54.7)			
Ovulation stimulation protocol	66 (14.2)	15 (11.8)		15 (12.8)	7 (13.2)			
Biochemical pregnancy rate, n (%)	234 (50.4)	33 (26)	< 0.001	48 (41)	7 (13.2)	< 0.001	0.069	0.060
Clinical pregnancy rate, n (%)	217 (46.8)	31 (24.4)	< 0.001	45 (38.5)	6 (11.3)	< 0.001	0.107	0.048
Live birth rate, n (%)	188 (40.5)	19 (15)	< 0.001	42 (35.9)	5 (9.4)	< 0.001	0.361	0.320
Spontaneous abortion rate, n (%)	24 (11.1)	10 (32.3)	0.001	2 (4.4)	1 (16.7)	0.319	0.272	0.646
Preterm birth rate, n (%)	21 (11.2)	1 (5.3)	0.700	6 (14.3)	1 (20)	0.571	0.597	0.380

*P*^*a*^: A1 vs. A2; *P*^*b*^: B1 vs. B2; *P*^*c*^: A1 vs. B1; *P*^*d*^: A2 vs. B2; Mann–Whitney U-test was used for the statistics of FSH, E2, LH.

*BMI* body mass index, *FSH* Follicle-stimulating hormone, *LH* luteinizing hormone, *E2* estradiol, *STP* surgical termination of pregnancy.

#### Stratified studies were performed based on the number of previous surgical termination of pregnancys

We divided the STP group into two subgroups based on the number of previous STPs; namely, C1 = 1-time abortion and C2 =  ≥ 2-times groups. The basic information and FET pregnancy outcomes of the two subgroups were compared, and the results displayed that the age and BMI of women in the C2 group were significantly higher than those in the C1 group. The endometrial thickness of women in the C2 group was significantly thinner than that in the C1 group. The biochemical pregnancy rate, clinical pregnancy rate, and live birth rate of women in the C2 group were significantly lower than those in the C1 group, the differences were statistically significant (P ≤ 0.01), the statistical power of the biochemical pregnancy rate was 82.48% and the clinical pregnancy rate was 83.1% and the live birth rate was 94.01%. As illustrated in Table 4.

**Table 4:** Comparison of basic conditions and FET pregnancy outcomes in subgroup analysis according to different surgical termination of pregnancy times.

Variable	C1 group n = 120	C2 group n = 50	*P* value
Age (years)	31.39 ± 4.52	33.96 ± 4.17	0.001
< 35 years	91 (75.8)	26 (52)	0.002
≥ 35years	29 (24.2)	24 (48)	
Duration of infertility (years)	3.89 ± 2.94	4.67 ± 3.55	0.172
BMI (kg/m^2^)	22.77 ± 3.44	24.1 ± 2.96	0.018
Endometrial thickness (mm)	10.64 ± 2.15	9.98 ± 1.72	0.038
Endometrial classification, n (%)			
A	10 (8.3)	2 (4)	0.377
B	103 (85.8)	47 (94)	
C	7 (5.8)	1 (2)	
Day 3 FSH (mIU/mL)	7.05 ± 2.23	8.17 ± 3.63	0.093
Day 3 LH (mIU/mL)	5.08 ± 3.55	4.42 ± 2.2	0.265
Day 3 E2 (pmol/L)	173.52 ± 126	165.52 ± 139.84	0.743
Number of embryos transferred	1.51 ± 0.53	1.5 ± 0.58	0.928
Embryo stage, n (%)			
Cleavage stage	96 (80)	44 (88)	0.213
Blastocyst stage	24 (20)	6 (12)	
Endometrial preparation protocol, n (%)			
Natural cycle protocol	38 (31.7)	17 (34)	0.758
Hormone replacement protocol	65 (54.2)	28 (56)	
Ovulation stimulation protocol	17 (14.2)	5 (10)	
Biochemical pregnancy rate, n (%)	46 (38.3)	9 (18)	0.010
Clinical pregnancy rate, n (%)	43 (35.8)	8 (16)	0.010
Live birth rate, n (%)	41 (34.2)	6 (12)	0.003
Spontaneous abortion rate, n (%)	2 (4.7)	1 (12.5)	0.407
Preterm birth rate, n (%)	6 (14.6)	1 (16.7)	1

Mann–Whitney U-test was used for the statistics of FSH, E2, LH, and antral follicle counts.

*BMI* body mass index, *FSH* follicle-stimulating hormone, *LH* luteinizing hormone, *E2* estradiol.

### Binomial logistic regression

#### Binomial logistic regression analysis of surgical termination of pregnancy and FET cycle clinical pregnancy rate

Confounding factors that influence clinical pregnancy include: age, BMI, number of embryos transferred, embryo stage, endometrial thickness, endometrial classification, duration of infertility, previous STP, and endometrial preparation protocol were incorporated into the regression equation, and the results demonstrated that: compared with women who had not previously undergone STP, previous STP was an influential factor in the decrease of clinical pregnancy rate during the FET cycle (OR = 0.614, 95% CI 0.413–0.911, P = 0.016). See Table 5 for details.

**Table 5:** Binomial logistic regression analysis of the influence of previous surgical termination of pregnancy on FET cycle clinical pregnancy rate.

		Control group	B value	*OR* value	*OR* 95% CI	*P* value
Age	≤ 30	35–42	1.199	3.316	1.979–5.557	< 0.001
	30–35	35–42	1.12	3.064	1.828–5.137	< 0.001
BMI			− 0.064	0.938	0.894–0.985	0.010
Number of embryos transferred			0.525	1.691	1.24–2.307	0.001
Embryo stage	Blastocyst stage	Cleavage stage	0.798	2.221	1.511–3.266	< 0.001
Endometrial thickness			− 0.010	0.990	0.914–1.072	0.797
Endometrial classification	A	C	− 0.148	0.862	0.419–1.773	0.687
	B	C	− 0.587	0.556	0.222–1.391	0.210
Duration of infertility			0.002	1.002	0.949–1.058	0.944
Previous STP	STP	Non-STP	− 0.488	0.614	0.413–0.911	0.016
Endometrial preparation protocol	Natural cycle protocol	Ovulation stimulation protocol	− 0.361	0.697	0.436–1.113	0.131
	Hormone replacement protocol	Ovulation stimulation protocol	− 0.457	0.633	0.378–1.062	0.083

Factors included in the regression equation included: age, BMI, number of embryos transferred, embryo stage, endometrial thickness, endometrial classification, duration of infertility, previous STP, and endometrial preparation protocol.

*BMI* body mass index, *STP* surgical termination of pregnancy.

#### Binomial logistic regression analysis of surgical termination of pregnancy and FET cycle live birth rate

We also adjusted the confounding factors influencing the live birth rate: age, BMI, number of embryos transferred, embryo stage, endometrial thickness, endometrial classification, duration of infertility, previous STP, and endometrial preparation protocol were incorporated into the regression equation. The effect of the previous STP on the live birth rate was not statistically significant (OR = 0.745, 95% CI 0.495–1.122, P = 0.159). See Table 6 for details.

**Table 6:** Binomial logistic regression analysis of the influence of previous surgical termination of pregnancy on FET cycle live birth rate.

		Control group	B value	*OR* value	*OR* 95% CI	*P* value
Age	≤ 30	35–42	1.78	5.931	3.135–11.22	< 0.001
	30–35	35–42	1.605	4.976	2.622–9.441	< 0.001
BMI			− 0.051	0.951	0.904–0.999	0.047
Number of embryos transferred			0.524	1.69	1.22–2.339	0.002
Embryo stage	Blastocyst stage	Cleavage stage	0.826	2.284	1.541–3.387	< 0.001
Endometrial thickness			− 0.008	0.992	0.914–1.078	0.858
Endometrial classification	A	C	− 0.315	0.73	0.351–1.517	0.398
	B	C	− 0.637	0.529	0.207–1.353	0.184
Duration of infertility			− 0.003	0.997	0.94–1.057	0.915
Previous STP	STP	Non− STP	− 0.294	0.745	0.495–1.122	0.159
Endometrial preparation protocol	Natural cycle protocol	Ovulation stimulation protocol	− 0.369	0.691	0.429–1.114	0.130
	Hormone replacement protocol	Ovulation stimulation protocol	− 0.534	0.586	0.344–0.999	0.050

Factors included in the regression equation included: age, BMI, number of embryos transferred, embryo stage, endometrial thickness, endometrial classification, duration of infertility, previous STP, and endometrial preparation protocol.

*BMI* body mass index, *STP* surgical termination of pregnancy.

#### Binomial logistic regression analysis of surgical termination of pregnancy and FET cycle live birth rate

To test whether the number of previous STPs was a risk factor for FET pregnancy outcomes. In addition to the number of STPs, the regression model included age and BMI as covariates. The results suggested that an increase in the number of prior STPs relative to only 1-time abortion was an independent risk factor in reducing clinical pregnancy rate and live birth rate (OR = 0.399, 95% CI 0.162–0.982, p = 0.046; OR = 0.32,95% CI 0.119–0.857, p = 0.023). See Table 7 for details.

**Table 7:** Binomial logistic regression analysis of the influence of different numbers of previous surgical terminations of pregnancy on clinical pregnancy outcomes in FET cycles.

			Control group	B value	*OR* value	*OR* 95% CI	*P* value
Clinical pregnancy rate	Age	≤ 30	35–42	1.177	3.245	1.098–9.592	0.033
		30–35	35–42	1.571	4.812	1.785–12.97	0.002
	BMI			− 0.012	0.988	0.89–1.098	0.828
	Number of previous STP	≥ 2-times abortion	1-time abortion	− 0.919	0.399	0.162–0.982	0.046
Live birth rate	Age	≤ 30	35–42	1.312	3.712	1.184–11.643	0.025
		30–35	35–42	1.568	4.795	1.658–13.868	0.004
	BMI			− 0.03	0.971	0.87–1.083	0.595
	Number of previous STP	≥ 2-times abortion	1-time abortion	− 1.14	0.32	0.119–0.857	0.023

Factors included in the regression equation included: age, BMI and number of previous STP.

*BMI* body mass index, *STP* surgical termination of pregnancy.

## Discussion

In this retrospective study, we investigated whether previous STP would affect pregnancy outcomes in the FET cycle. The final analysis demonstrated that previous STP was an independent factor contributing to the decline in FET cycle clinical pregnancy rates. Although the effect on live birth rates did not reach statistical significance, our data actually strongly tilts toward the conclusion that STP may have had an adverse effect on the live birth rate.

There were few studies about the effects of ITP on the outcome of assisted pregnancy. The studies reported so far were focused on fresh cycles. It is generally believed that previous ITP can lead to the deterioration of the outcome of fresh cycle pregnancy, decline of clinical pregnancy rate and the live birth rate with the increase of spontaneous abortion rate^15^, and this trend will become severe with the rise in the number of ITPs^16^, consistent with our results.

However, this study could not discover that a previous STP caused a significant increase in the spontaneous abortion rate of the FET cycle. Previous studies have demonstrated that STP can damage the structure of the female endometrium^18^**.** STP may also change the secretory function of the endometrium and the secretion of inflammatory factors, thus interfering with the combination of uterus and embryo, leading to the increase of spontaneous abortion^16^. In our opinion, the reason there was no significant difference in spontaneous abortion rate between the two groups in the FET cycle was due to the careful endometrium preparation, and the freeze–thaw process of embryos in the FET cycle may also play a particular role in selecting embryos.

Previous STP was associated with a significant decrease in endometrial thickness compared to patients who had not previously undergone a STP. Furthermore, endometrial thickness will become thinner and thinner with increased abortions^19^**.** A similar situation was discovered in our research. We found that despite the careful endometrial preparation required by the FET, endometrial thickness was thinner in women in the miscarriage group than in women of the same age who never had a STP. Simultaneously, with the increase in abortions, endometrial thickness also revealed a decreasing trend. Relevant studies have depicted that in addition to the direct damage to the endometrium, the surgical operation during an abortion will further reduce the blood flow to the myometrium and cause direct damage to the endometrial stem cells^19,20^. Embryo implantation is a process in which the embryo and maternal endometrium recognize, accommodate, and interact. Endometrial receptivity is closely related to pregnancy outcomes^21^**.** The commonly used indicators for judging endometrial receptivity include endometrial volume, endometrial thickness, and endometrial blood flow^22^. In our study, we found that STP caused a decrease in clinical pregnancy rate, but no correlation was found between endometrial thickness and clinical pregnancy rate in multiple regression analysis. We analyzed that the reason for this situation may be that although women who have undergone STP have thinner endometrium, but, STP may cause endometrial fibrosis and abnormal blood vessel distribution^10,23,24^, thereby affecting endometrial function, and these reasons may be the key to the decline in clinical pregnancy rate.

Some studies have shown that endometrial dysfunction was the main factor in fertility decline in obese women^25,26^. This study also found this phenomenon. We found that BMI was an independent factor influencing clinical pregnancy rate,and live birth rate. An increase in BMI negatively affected clinical pregnancy rates, live birth rates. Therefore, keeping the patient's weight within the normal range during FET is imperative.

When we stratified by age, there was no significant difference in basal endocrine levels between patients with a history of STP and controls of the same age group. This is similar to the previous results^15,16^. This suggested that STP will not significantly impact the endocrine function of the ovary.

Presently, the primary abortion methods encompass surgical and medication procedures. Globally, approximately half of all abortions are conducted through medication^27^. Conditional upon the absence of relevant contraindications, medication abortion predominantly relies on the combination of mifepristone and misoprostol, as endorsed by WHO guidelines for safe abortion care. Compared to surgical abortion, complications following medication abortion are markedly rare, with main adverse events including infection, retained pregnancy, prolonged pain, extended vaginal bleeding, or failed abortion^28,29^. Our study indicates that a history of surgical abortion may pose a potential risk for pregnancy outcomes in FET cycles. However, regrettably, the impact of medication abortions on future pregnancies and their potential contribution to assisted reproduction outcomes remain unclear. A comparative study assessing long-term fertility and pregnancy outcomes between women who underwent medication and surgical abortion demonstrated similar late spontaneous conception rates (97.7%), comparable cumulative 12- and 24-month pregnancy rates (60% and 80%, respectively), and no significant differences in subsequent live birth rates (85.2 vs. 88.2%, P = 0.72)^30^. Another investigation among women undergoing fresh-cycle IVF revealed that those with a history of medication abortion exhibited similarities to participants with no abortion history in terms of embryo implantation, clinical pregnancy rate, live birth rate, and spontaneous abortion rate. However, participants with a history of medication abortion did display a significantly lower endometrial thickness compared to those with no abortion history^16^. It is important to note that these findings do not assert the superiority of medication abortion, and more research is still needed to confirm the existence of this association.

We reviewed and analyzed the FET data of our center from the past seven years by referring to the medical record system in the hope of answering the question of whether previous STP will affect the FET cycle? Our study comprehensively analyzed the impact of previous STP on the frozen-embryo cycle. All data were from the case system with high reliability. However, this study also has its shortcomings. First, due to the limitations of objective conditions, we could not learn the specific information of each patient undergoing surgery: the intraoperative situation, the effect of the operation, whether the operation institution was regular, the proficiency of the surgeon, the treatment after the procedure, and whether there were complications after the operation. Second, this study is a retrospective study with a conservative conclusion, which needs to be verified by prospective studies. Moreover, this study was a single-center study, which could have affected the reliability of the conclusion. It should also be noted that the sample size of the included studies is limited, and we need a larger sample size and the participation of more research institutions to confirm the association and the extent of the effect.

## Conclusion

In summary, this study found that previous surgical abortion may be one of the adverse factors for FET cycle clinical pregnancy rates. Although the effect on live birth rates did not reach statistical significance, our data actually strongly tilts toward the conclusion that STP may have had an adverse effect on the live birth rate, but we need a larger sample size is needed to confirm this association and the degree of impact. For women who have had a previous STP, multiple STP may be one of the reasons for the decline in clinical pregnancy and live birth rates in this group. Given that the reproductive health effects of STP are reported in numerous studies, we strongly recommend that women who are considering STP, as well as those with a history of STP who seek help to conceive, should be informed of the potential negative effects on future pregnancies, including FET pregnancies. At the same time, we should be more active in advocating and publicizing the correct contraceptive methods as far as possible to reduce the occurrence of ITP.

## Data Availability

The datasets used and/or analyzed during the current study are available from the corresponding author on reasonable request.
